# Role of Counterions in the Structural Stabilisation of Redox‐Active Metal‐Organic Frameworks**

**DOI:** 10.1002/chem.202203843

**Published:** 2023-02-13

**Authors:** M. J. Golomb, K. Tolborg, J. Calbo, A. Walsh

**Affiliations:** ^1^ Department of Materials Imperial College London Exhibition Road London SW7 2AZ UK; ^2^ Instituto de Ciencia Molecular Universidad de Valencia 46890 Paterna Spain

**Keywords:** ab-initio calculations, density functional theory, hybrid materials, materials science, metal-organic frameworks

## Abstract

The crystal structures of metal‐organic frameworks (MOFs) are typically determined by the strong chemical bonds formed between the organic and inorganic building units. However, the latest generation of redox‐active frameworks often rely on counterions in the pores to access specific charge states of the components. Here, we model the crystal structures of three layered MOFs based on the redox‐active ligand 2,5‐dihydroxybenzoquinone (dhbq): Ti_2_(Cl_2_dhbq)_3_, V_2_(Cl_2_dhbq)_3_ and Fe_2_(Cl_2_dhbq)_3_ with implicit and explicit counterions. Our full‐potential first‐principles calculations indicate that while the reported hexagonal structure is readily obtained for Ti and V, the Fe framework is stabilised only by the presence of explicit counterions. For high counterion concentrations, we observe the formation of an electride‐like pocket in the pore center. An outlook is provided on the implications of solvent and counterion control for engineering the structures and properties of porous solids.

## Graphical abstract

Revealing the role of counterions: Redox‐active metal‐organic frameworks often rely on counterions to access specific charge states of the components. We show, through a quantum chemical investigation, that the counterion can play an important steric role in the structural stabilisation of such frameworks beyond pure charge balancing effects.

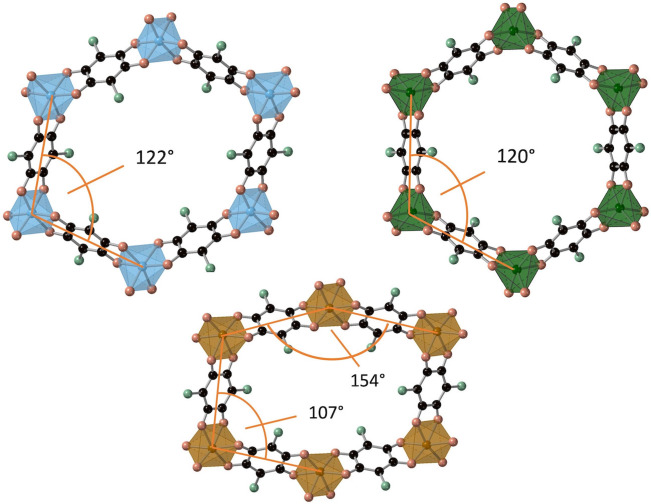

## Introduction

Metal‐organic frameworks (MOFs) are a class of hybrid materials that, since their initial description,[^1^, ^2^] have sparked interest in various fields of research including gas storage^[3]^ and separation,^[4]^ sensing,^[5]^ catalysis,[^6^, ^7^] drug delivery,^[8]^ and more recently in energy‐related devices such as supercapacitors[^9^, ^10^] and batteries.[^11^, ^12^]

MOFs are formed of metal ions or clusters connected by organic ligands, leading to a great number of crystalline and amorphous structures with varying composition and topology. Their modular nature offers opportunities regarding tunability for target properties of synthesised materials via the exchange of building blocks or incorporation of guest molecules.[^13^, ^14^, ^15^, ^16^] Inherently, MOFs are expected to be electrical insulators or wide‐gap semiconductors due to the energy and symmetry mismatch between the frontier orbitals of common metal nodes and organic ligands.^[17]^ In the last decade, however, interest in tuning the electronic properties of MOFs has risen, leading to frameworks with conductivity values up to 10^3^ S/cm,[^17^, ^18^, ^19^] which is comparable to commercially used organic semiconductors. This makes conductive MOFs interesting candidates for future electronic devices.^[20]^

Despite these achievements, the underlying principles of charge transport in MOFs are not well articulated.^[21]^ Several strategies have been employed to enhance their conductivity, such as redox‐matching of the building blocks, the use of mixed‐valence metal nodes and ligands, or the introduction of conducting guests into the pore.[^22^, ^23^, ^24^, ^25^, ^26^]

**Figure 1:**
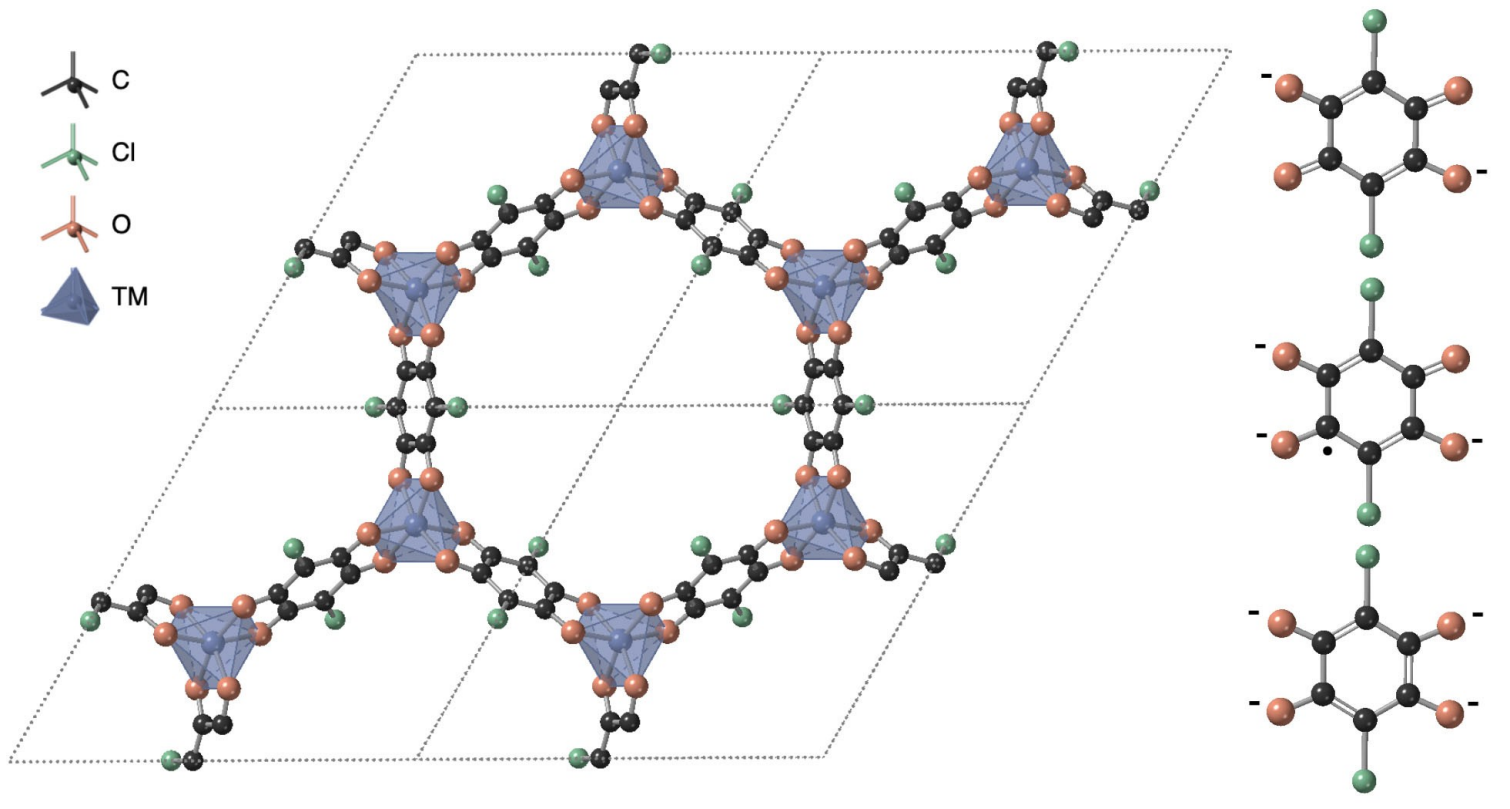
Crystal structure of M_2_(Cl_2_dhbq)_3_ oriented through the *c* axis, where M is a transition metal (Ti, V, Fe). The accessible 2^−^, 3^−^ and 4^−^ charge states of the ligand are also shown.

Engineering high‐performing MOFs for a desired application involves searching the vast configuration space of metal nodes, linkers and ligands. Among the large list of redox‐active ligands used to promote conductivity in porous frameworks, dihydroxybenzoquinone (dhbq) and its derivatives stand promising to engender mixed‐valence MOFs due to their ability to exist in three different redox states through aromatic‐quionid‐aromatic transitions (Figure 1).^[27]^ The 3^−^ charge state is a radical and thus carries a spin compared to the diamagnetic 2^−^ and 4^−^ states. The π*‐orbitals of the ligand are energetically matched with transition metal 3*d*‐orbitals, enabling effective metal‐ligand coordination and long‐range conjugated pathways along the framework struts for efficient charge transport.^[28]^

The magnetoelectronic properties of three MOFs using dhbq as their ligand have been investigated experimentally.[^29^, ^30^] The conductivities of Ti_2_(Cl_2_dhbq)_3_, Fe_2_(Cl_2_dhbq)_3_ and V_2_(Cl_2_dhbq)_3_ were found to be 2.7×10^−3^ S/cm, 1.4×10^−2^ S/cm and 0.45 S/cm, respectively. These are high values compared to other MOFs, especially V_2_(Cl_2_dhbq)_3_ which is one of the most conducting frameworks based on oxo‐donor linkers reported to date. Optical spectroscopy indicated a high degree of electronic delocalisation in V_2_(Cl_2_dhbq)_3_, which led to a classification as a Robin‐Day class III mixed‐valence compound, whereas Ti_2_(Cl_2_dhbq)_3_ and Fe_2_(Cl_2_dhbq)_3_ were classified as class II. Due to the delocalisation, a certain assignment of the charge states of the building blocks in V_2_(Cl_2_dhbq)_3_ was not possible and remains an open question. Band transport analysis from density functional theory (DFT) calculations on single layers of the MOFs indicated linear dispersion in both spin channels for V_2_(Cl_2_dhbq)_3_ and in one spin channel for Ti_2_(Cl_2_dhbq)_3_, classifying those MOFs as Dirac metals and Dirac half metals, respectively.^[31]^

While it is known experimentally that counterions can have a significant effect on the framework structures,^[32]^ the mechanism is largely unexplored. No theoretical study of the stabilisation via counterions and their effect on the redox state of the MOF building blocks has been reported to our knowledge. In this work, we analyse the crystal structures of the three MOFs Ti_2_(Cl_2_dhbq)_3_, Fe_2_(Cl_2_dhbq)_3_ and V_2_(Cl_2_dhbq)_3_ by means of first‐principles calculations. Our analysis indicates that the favoured framework topology is highly dependent on the explicit presence of the counterion. In particular, the experimentally observed hexagonal phase of Fe_2_(Cl_2_dhbq)_3_ is predicted to be stable only upon the introduction of counterions in the framework pore. For counterion concentrations above the suggested stoichiometry, the additional electron donation does not alter the oxidation state of the framework but forms a localized state in the pore center, similar to an electride crystal^[33]^ or a hydrated electron.^[34]^ These results emphasise the importance of the solvent/counterions as a variable when designing and characterizing redox‐active hybrid frameworks.

## Results & Discussion

### Experimental crystal structures

All variants of M_2_(Cl_2_dhbq)_3_ have been reported to exist in the trigonal space group $P\bar{3}1m$ . The crystal structure has been described as two‐dimensional layered honeycomb sheets, with one‐dimensional pore channels running perpendicular to the layers (Figure 1). Reported intralayer metal‐to‐metal distances vary from 7.73 to 7.84 Å, with pore widths of up to 15.7. Interlayer distances of the experimentally synthesized compounds range from 8.65 to 8.69 Å. All of these structures are reported to be perfectly eclipsed forming one‐dimensional pore channels perpendicular to the layer sheet plane.[^29^, ^30^] Fe_2_(Cl_2_dhbq)_3_ has however also been reported in a three‐dimensional phase in the cubic space group $I\bar{4}3d$ as two interpenetrated frameworks of opposing chirality.^[35]^

### Implicit electrostatic charge compensation

As a first step, the crystal structure of competing spin configurations was investigated for all three materials using a compensating background charge to achieve an electroneutral framework with the assigned oxidation states.

**Figure 2:**
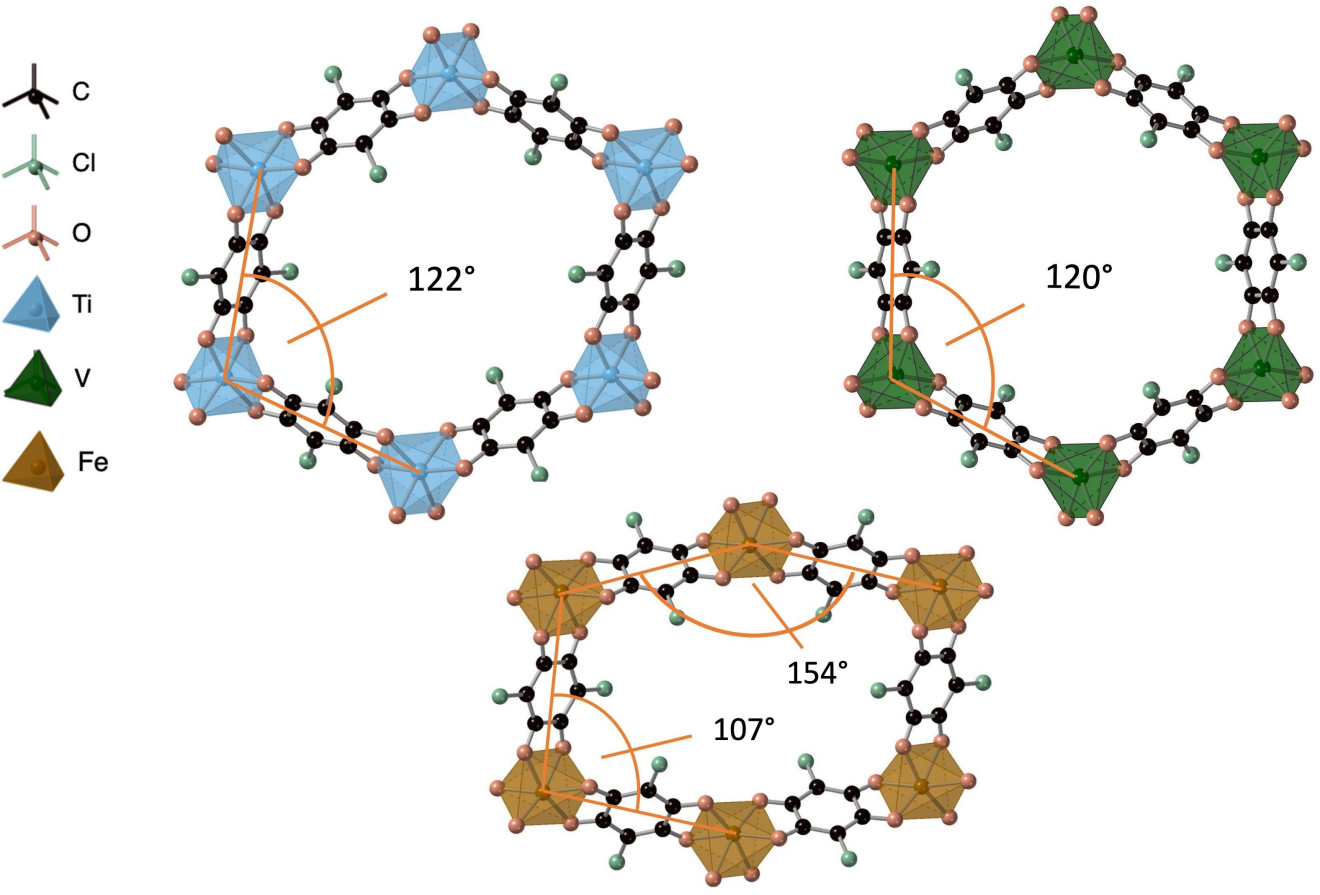
Optimised crystal structures of the three M_2_(Cl_2_dhbq)_3_ frameworks including an implicit background charge of 2^−^ per unit cell. The M-M-M angle in an ideal hexagonal framework is 120°.

Since the Ti based structure was reported as [Ti^IV^_2_(Cl_2_dhbq^3−^)_2_(Cl_2_dhbq^4−^)]^2−^, we initialised calculations in the antiferromagnetic and ferromagnetic configurations of the radical spins. As Ti(IV) has a d^0^ electronic configuration, no direct contribution ob e magnetism is expected. The antiferromagnetic alignment was found ob e only 2 meV more stable per atom than a ferromagnetic counterpart, which makes it instructive to consider both phases due to thermal accessibility at room temperature. This is in agreement with experimental reports of paramagnetic ligand spins with antiferromagnetic interaction at low temperatures. A similar result was obtained for V_2_(Cl_2_dhbq)_3_, where spins were initialised following the experimentally proposed composition of [V^III^V^IV^(Cl_2_dhbq^3−^)_3_]^2−^. This corresponds to V d^2^ and d^1^ configurations. This assignment was made tentatively by experiment, which is why the two metal centers were also studied with initialization of V^III^_2_ and V^IV^_2_. Spin states were found to be highly competitive, with N=N↑-N↓= 2 being favoured by less than 1 meV per atom (see Table S1), which is far less than the available thermal energy at room temperature. The optimised structures agree with prior experiments in terms of similar intralayer metal‐to‐metal distances (7.77 for Ti and 7.67 for V) and interlayer distance (8.67 for Ti and a slightly deviating 8.01 Å for V). The Ti‐based structure however differs strongly from the reported layer stacking: instead of being eclipsed, subsequent layers are displaced ($\alpha \approx 115^{\circ }$ , $\beta \approx 78^{\circ }$ ) by d≈2.9 Å), as seems to be common in other layered frameworks.^[36]^ For [Fe^III^_2_(Cl_2_dhbq^2−^)(Cl_2_dhbq^3−^)_2_]^2−^, no combination of spin initialization or variation of the atomic relaxation parameters led to the reported hexagonal structure. Instead, the trifold symmetry around the metal node is found to be broken, leading to angles deviating from their quasi‐hexagonal values of around 122° to 107 and 154°, respectively (Figure 2). This behaviour has recently been reported for similar MOF structures,^[37]^ but has to our knowledge not been observed experimentally for this system.

**Figure 3:**
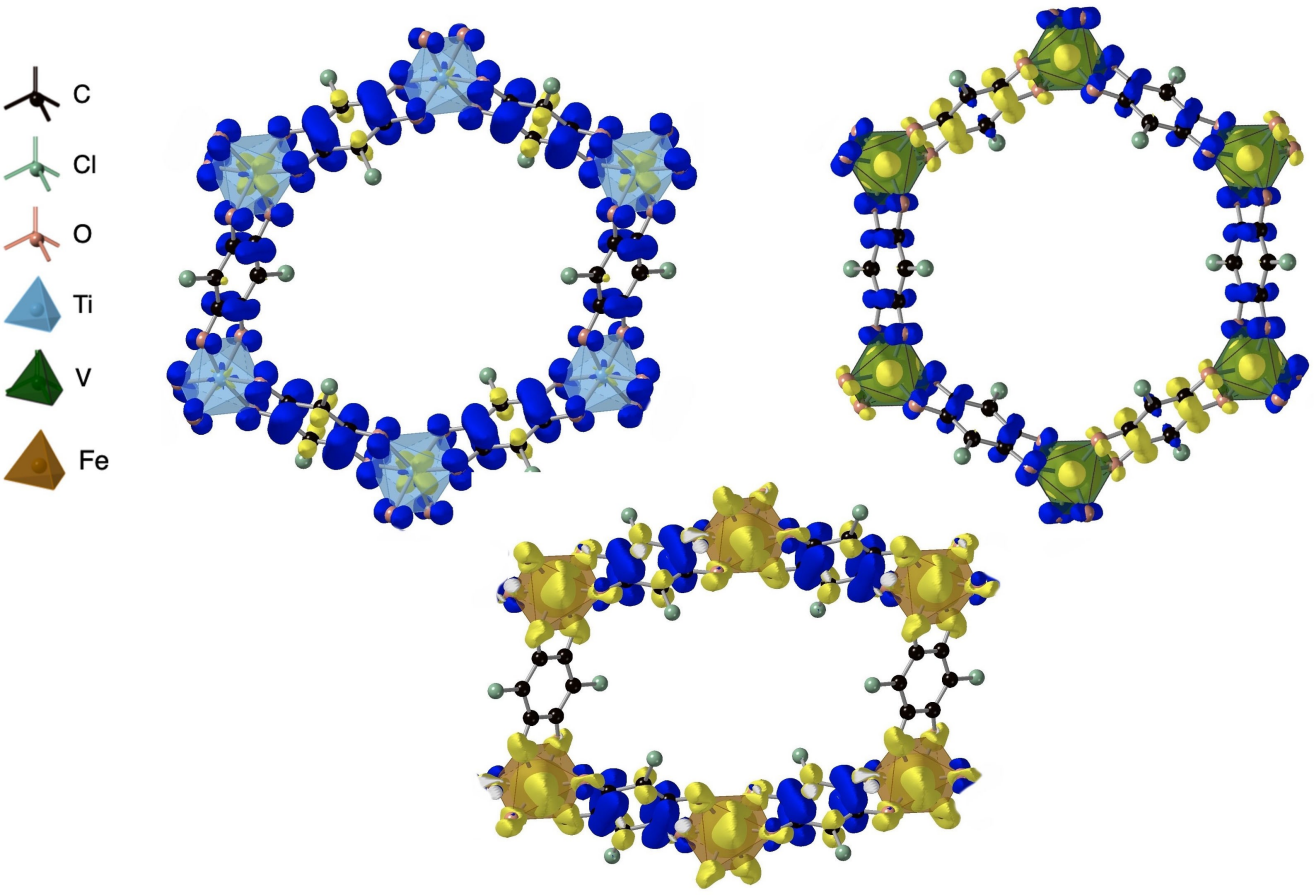
Excess spin density of Ti_2_(Cl_2_dhbq)_3_, V_2_(Cl_2_dhbq)_3_ and Fe_2_(Cl_2_dhbq)_3_ considering a homogeneous background charge. Here a mixed valence solution of Ti(IV) d^0^ and Ti(III) d^1^ cations is found for Ti_2_(Cl_2_dhbq)_3_, while Fe(III) *d* ^5^ is found for Fe_2_(Cl_2_dhbq)_3_.

To explain the different structural results obtained for Ti, V and Fe, we visualize the excess spin density (ρ↑-ρ↓ ) in each system. This provides evidence for the oxidation states of ligand and metal node in the obtained geometries. As can be seen in Figure 3, there is a stark difference in the ground state electron distributions for the different MOFs when considering a homogeneous background charge.

In the Ti‐based framework, all ligands have a similar oxidation state and thus maintain trifold symmetry around the metal node. This is achieved by Ti adopting a mixed valence configuration with both Ti(IV) and Ti(III),^[38]^ while the ligands are best described as Cl_2_dhbq^3−^. The antiferromagnetic coupling of the metal spin to the ligand spins might be able to explain the deviation from the expected magnetic moment seen in experiment.^[29]^ This is similar to the V‐based material, where one of the ligand spins is antiferromagnetically coupled to the others. The expected V mixed valency is however not confirmed, as both metal atoms are seemingly found in the same oxidation state. A Mulliken analysis assigns each site a spin of N= 1.3, in accordance with the experimental description of this material not being fully described with a traditional assignment of redox states. The aforementioned trifold symmetry around the metal node is clearly broken in the Fe structure, leading to a deviation from the hexagonal phase. The experimental oxidation state assignment [Fe^III^_2_(Cl_2_dhbq^2−^)(Cl_2_dhbq^3−^)_2_]^2−^ is confirmed in this case, revealing an antiferromagnetic coupling of the high spin metals and two ligands.

### Explicit counterion charge compensation

**Figure 4:**
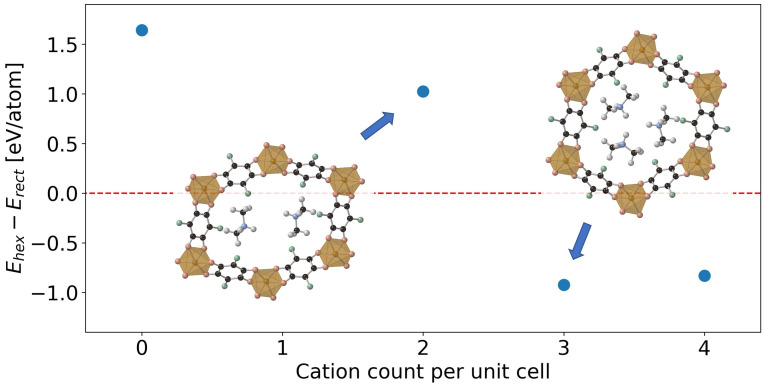
Total energy difference per atom between hexagonal and rectangular polymorphs as a function of the presence of an explicit charge balancing cation, i. e. (Me_2_NH_2_)_*x*_Fe_2_(Cl_2_dhbq)_3_ where x=0-4 (0 corresponds to an implicit background charge).

The deviation between the observed and predicted structure of Fe_2_(Cl_2_dhbq)_3_ prompted further consideration. A comparison of the energy between the computationally relaxed structure and constrained optimisation of the experimentally reported hexagonal phase revealed the latter to be more than 1 eV per atom higher in energy (Figure 4, x=0 ). This anomalously large energy difference is due to a distinct electronic solution with spin localization on one ligand only, despite an initialization of spins on two different ligands (Figure S1).

The next step is to include an explicit description of the charge balancing counterions. In previous work, the counterions were assumed to reside in the pore based on a stoichiometry of (Me_2_NH_2_)2Fe_2_(Cl_2_dhbq)_3_, where Me_2_NH_2_ is dimethylamine generated from decomposition of the dimethylformamide (DMF) solvent.^[29]^ A relaxation of this crystal structure including two counterions per unit cell did not result in a hexagonal framework, even when considering multiple starting positions for the counterions to exclude the possibility of a shallow potential energy surface leading to non‐hexagonal local minima (Figure 4, x=2 ).

We also investigated the possibility of stabilisation of the hexagonal phase via superstructure effects with two counterions per unit cell. While it is only possible to consider one orientation of two cations in a unit cell, different orientations of cations in neighbouring cells can be considered in a supercell. Due to computation limitations, we employed a 3×1×1 supercell expansion, allowing for orientational variation along one axis. An unconstrained crystal structure relaxation leaves the hexagonal structure of the unit cell intact in this case (Figure S2). The total energy per atom of the hexagonal supercell is 10 meV lower per atom than that of the hexagonal unit cell, but still considerably higher than the rectangular cell, suggesting the static supercell structure to be a high energy metastable state. A calculation starting from the rectangular geometry was not performed since the addition of further degrees of freedom would only lead to an energy lowering and thus to no change in the ranking of energy per atom of the considered geometries.

### Hexagonal stabilisation mechanism

In the static models, inclusion of a third counterion in the unit cell leads to a stable hexagonal phase (Figure 4, x=3 ). A variety of metastable or unstable phases were probed by constraining the unit cell angles to the values obtained in their respective stable phases, while varying the counterion count per unit cell.

**Figure 5:**
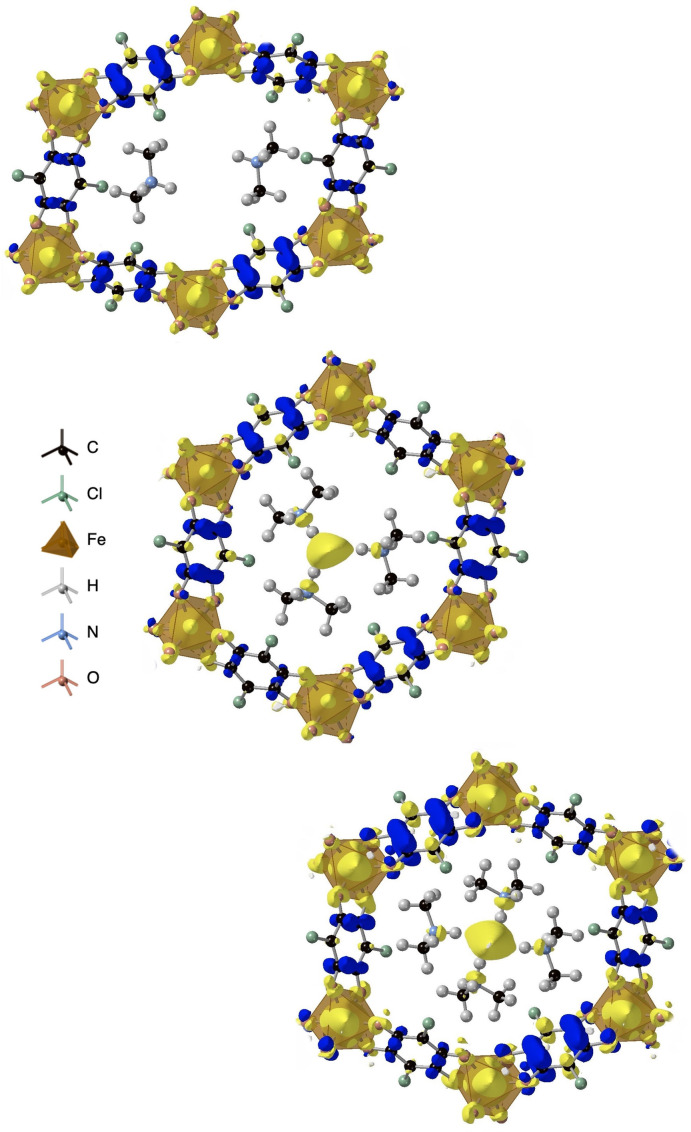
Excess spin density of (Me_2_NH_2_)_*x*_Fe_2_(Cl_2_dhbq)_3_ in their relaxed crystal structures. The x=2 rectangular phase (top) transforms to the hexagonal framework for x=3 (middle) and x=4 (bottom).

This behaviour can be explained by spin density analysis of the Fe framework. When considering explicit counterions, the oxidation states of the ligand in the framework (Me_2_NH_2_)_*x*_Fe_2_(Cl_2_dhbq)_3_ change to a more even distribution across all three ligands, similar to what is observed in the Ti and V frameworks (Figure 5). When x=2 , the ligand that formerly had no radical acquires spin density, moving closer to the experimentally described distribution of 2/3 of an electron on each ligand,^[30]^ although it does not appear to be uniform across all ligands and the factors responsible for breaking the trifold symmetry without counterions still dominate. Upon consideration of a third ion, the hexagonal phase emerges as the groundstate. An explanation for this is sterics: to achieve trifold symmetry, a third counterion is necessary. This does however not result in a change in charge state of the framework, but instead leads to an electride‐like state with an electron localised in the pore center. This resistance to change in charge state of the framework is in agreement with the experimental charge assignment of the framework via spectroscopy.^[30]^ It also persists when considering higher counterion concentrations in the framework. This is seemingly different to the experimentally performed framework reduction by soaking the structure in cobaltocene, where experiment suggests three charge balancing cations in the pore to lead to a 3^−^ framework charge state,^[30]^ further pointing to the importance of explicit consideration of counterions in metal‐organic frameworks. It has to be pointed out that while our study does consider Me_2_NH_2_ explicitly, there is a possibility of charge compensation via other synthesis byproducts such as solvated metal cations, as previously noted by Ziebel et al.^[29]^ We furthermore note that our simulations are based on static models, so it is possible that a dynamic distribution of two counterions could be sufficient to stabilize a hexagonal phase, but nonetheless the explicit role of the counterion is critical.

## Conclusions

A systematic computational study of the ground state structure of the three two‐dimensional layered MOFs Ti_2_(Cl_2_dhbq)_3_, Fe_2_(Cl_2_dhbq)_3_ and V_2_(Cl_2_dhbq)_3_ has been conducted. Ground‐state spin configurations were determined and compared energetically. The oxidation states of the building blocks have also been resolved. For a direct comparison with experiment, it has to be noted that small cell calculations do not capture stacking faults that may have a significant impact on the structures of layered frameworks.^[39]^

The selection of solvent and counterions is not only important for templating and charge balancing, but also for their persistent role in determining the framework structure and symmetry – making it another parameter or building block of MOF chemistry and increasing their chemical space even further.^[40]^ The dependence on the choice of metal also shows that some MOFs are more structurally resilient to the influence of excess counterions.

Our findings also question a common practice in the creation of MOF structure‐property databases: the computational cleaning of pore species including solvent before data deposition. This will lead to erroneous structures and unphysical calculated properties for certain classes of MOFs, such as those studied here.

## Computational Methods

Crystal structure optimisation was performed based on atomic forces and unit cell stresses from density functional theory (DFT). A trust radius enhanced Broyden‐Fletcher‐Goldfarb‐Shanno algorithm^[41]^ was used to achieve forces of less than 0.01 eV/Å per atom, using the Lindh model matrix as an initial guess for the Hessian.^[42]^ The underlying Kohn‐Sham equations were solved using the all‐electron FHI‐Aims package.[^43^, ^44^, ^45^, ^46^, ^47^] We followed a two‐step approach for computational efficiency: (i) initial relaxation under periodic boundary conditions with the tier 1 basis set using the semi‐local PBEsol functional;^[48]^ (ii) further relaxation with the tier 2 basis set and the same functional. All calculations were performed spin unrestricted and include Tkatchenko‐Scheffler dispersion.^[49]^ Due to the many possible spin configurations and their subtle difference in energy, the final energies were calculated with the tier 2 basis set using the hybrid HSE06 functional^[50]^ with 25 % Hartree Fock exchange. Cation insertion into the pore and building of supercells was performed using crystalmaker.

## Conflict of interest

There are no conflicts of interest to declare.

1

## Supporting information

As a service to our authors and readers, this journal provides supporting information supplied by the authors. Such materials are peer reviewed and may be re‐organized for online delivery, but are not copy‐edited or typeset. Technical support issues arising from supporting information (other than missing files) should be addressed to the authors.

Supporting Information

## Data Availability

The data that supports the findings of this study is openly available at https://doi.org/10.5281/zenodo.7311089.
