# Supplementary material for: Role of Counterions in the Structural Stabilisation of Redox‐Active Metal‐Organic Frameworks
Source: Chemistry. 2023 Feb 13;29(16):e202203843. doi: 10.1002/chem.202203843 (PMC10946919; doi:10.1002/chem.202203843)
Supplement: Supplementary file 1 — Supporting Information [file CHEM-29-0-s001.pdf]

Chemistry–A European Journal

Supporting Information

Role of Counterions in the Structural Stabilisation of Redox-Active Metal-Organic Frameworks

M. J. Golomb, K. Tolborg, J. Calbo, and A. Walsh*

Framework	μ_B metal 1	μ_B metal 2	μ_B ligand 1	μ_B ligand 2	μ_B ligand 3	μ_B total	Energy above min [eV]
FeDHBQ	5	-5	1	-1	0	0	**0**
	5	-5	1	1	0	2	0.35
	5	5	-1	-1	0	8	0.10
	5	5	1	-1	0	10	0.63
	5	5	1	1	0	12	0.63
TiDHBQ	0	0	1	-1	0	0	0.46
	0	0	1	1	0	2	**0**
VDHBQ	2	-2	1	-1	0	0	1.16
	2	-1	1	-1	-1	0	0.01
	2	1	-1	-1	-1	0	0.01
	1	-1	1	-1	0	0	0.01
	2	-2	1	1	0	2	1.25
	2	2	-1	-1	0	2	1.18
	2	-1	1	1	-1	2	<0.01
	1	-1	1	1	0	2	<0.01
	1	1	1	-1	0	2	<0.01
	2	2	1	-1	0	4	1.40
	2	1	1	1	-1	4	0.19
	2	-1	1	1	1	4	0.19
	1	1	1	1	0	4	0.19
	2	2	1	1	0	6	1.36
	2	1	1	1	1	6	0.18

Table S 1 Total energy difference compared to the minimum energy configuration for all spin initialization considered

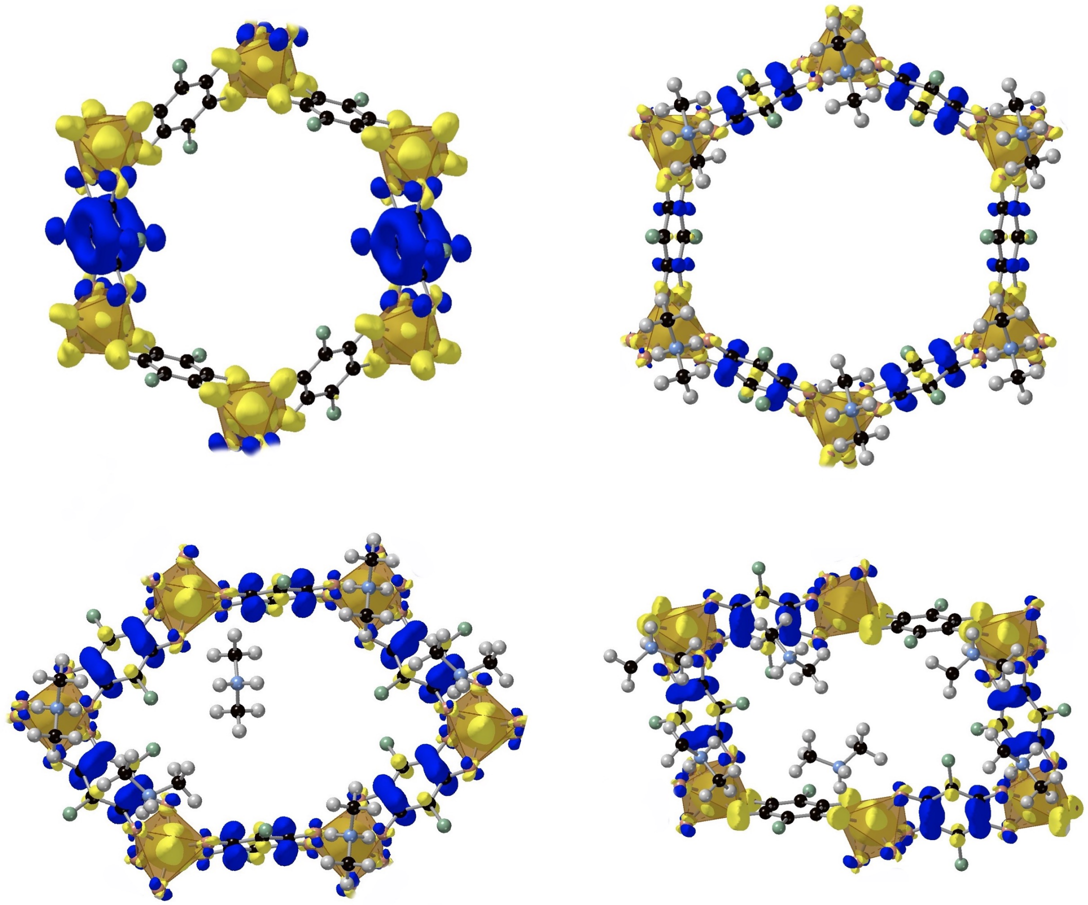

Figure S 1 Excess spin density of the constrained calculations initialized from the respective unstable phases. Top: hexagonal phase with implicit background charge (left) and with two counterions (right). Bottom: rectangular phase with three (left) and four (right) counterions per unit cell.

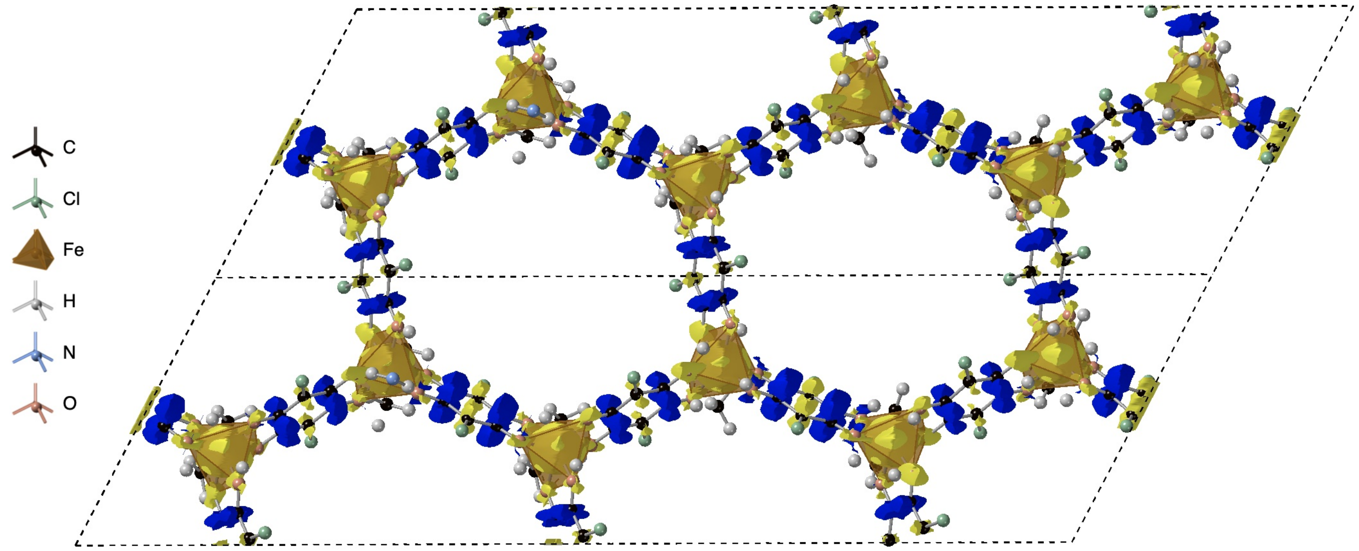

Figure S 2 Excess spin density of the 3×1 supercell including two counterions per unit cell.
